# Co-cultivation of human granulosa cells with ovarian cancer cells leads to a significant increase in progesterone production

**DOI:** 10.1007/s00404-023-06914-z

**Published:** 2023-01-18

**Authors:** Detlef Pietrowski, Martina Grgic, Isabella Haslinger, Julian Marschalek, Christian Schneeberger

**Affiliations:** 1 Department of Obstetrics and Gynecology, Medical University of Vienna, Spitalgasse 23, 1090 Vienna, Austria; 2 FH Campus Wien, University of Applied Science, Vienna, Austria

**Keywords:** Steroid hormone, Ovary, In vitro model, Cellular interaction, cAMP

## Abstract

**Purpose:**

In humans, granulosa cells (GCs) are part of the follicle and nourish the growing oocyte. GCs produce estrogen and, after ovulation, progesterone. They are embedded in a multicellular tissue structure of the ovary, which consists of a variety of different cell types that are essential for the physiological function of the ovary. However, the extent to which individual ovarian cell types contribute to overall functionality has not yet been fully elucidated. In this study, we aim to investigate the effects of co-culturing human granulosa cells with ovarian cancer cells on their progesterone and estrogen production in an in vitro model.

**Methods:**

After seeding, the cells were stimulated with 200 µM forskolin in DMEM for 72 h and the medium of the different cell culture experiments was collected. Subsequently, progesterone and oestradiol concentrations were determined using an Elisa assay.

**Results:**

Morphologically, it was striking that the cells self-organize and form spatially separated areas. Compared to culturing granulosa cells alone, co-culturing human granulosa cells together with the ovarian cancer cell line OvCar-3 resulted in a significant increase in progesterone production (20.3 ng/ml versus 50.2 ng/ml; *p* < 0.01).

**Conclusions:**

Using a simple in vitro model, we highlight the importance of cellular crosstalk between different ovarian cells in a complex cellular network and that it strongly influences granulosa cell hormone production. This could have potential implications for the procedure of transplanting endocrine tissues after cryopreservation, as it highlights the importance of survival of all cells for the functionality of the transplanted tissue.

## What does this study add to the clinical work

**Table:** 

The proposed in vitro model, using ovarian cells as an example, suggests that when ovarian tissue is re-transplanted, the cell types that are most severely damaged can determine the success or failure of the intervention. Furthermore, it indicates a remarkable influence of ovarian cancer cells on its cellular environment.	

## Introduction

The ovaries—as part of the female reproductive organs—represent a polymorphic complex structure consisting of a multitude of different cell types, each with different functions. To ensure full functionality of the ovary as an organ, all tissue and cell types must interact with each other in a finely orchestrated manner [1, 2].

An essential feature of a morphologically and physiologically intact ovary is the ability to produce sex steroid hormones under the control of gonadotropins. It is well established that granulosa cells (GC) and theca cells (TC) play a key role in this physiological process, being the sites of estrogen and progesterone production. For in vitro studies on ovarian hormone production, GC-based in vitro cell model systems have been developed and provide a valuable tool for an analysis of molecular pathways in hormone production [3, 4]. The major source of human GCs for in vitro studies is usually derived from patients undergoing artificial reproductive technologies (ART). However, these cells are accessible only in limited amounts, which make it difficult to perform widespread experiments related to detailed molecular analysis. Furthermore, and because of the therapy-specific gonadotropin-stimulation with ovulation induction, clinically obtained GCs are mostly luteinized. These GCs have a restricted life span with a slow proliferation rate, and they do not stay viable in vitro after many passages [3, 5]. In addition, primary GCs derived from specific patients may exhibit very high variability, which further complicates the achievement of reproducible results. Due to these problems, permanent cell models with primary GCs are difficult to establish. Therefore, in in vitro studies, human GC cell lines are often considered an attractive option [3, 6]. For example, the immortalized Granulosa cell lines KGN and HGL- 5 have been shown to be valuable in ovarian functional studies [7]. While the KGN cell line produces significant amounts of progesterone after stimulation with cyclic adenosine monophosphate (cAMP) inducing agents other cell lines do not. The KGN cell line was therefore considered particularly useful for in vitro studies of ovarian steroid hormone production.

Recently, it was shown that cell-to-cell contact is a pre-requisite to improve cellular vitality and that a specific microenvironment is necessary for promotion of endocrine function in various tissues including the ovary [8–10]. In addition, Fozzatti and co-authors have shown that cancer development and cancer progression depend on the mutual interaction between growing tumor cells and their surrounding cellular microenvironment [11].

In the ovary. the influence of other cells on GC triggered hormonal production has not been extensively studied. We hypothesize that GC surrounding cells may have an impact on hormone production of GCs. To address this question, we developed a simple two-cell model consisting of the human granulosa cell line KGN and the human carcinoma cell line OvCar-3 and analyzed the impact of this ovarian cell lines on hormone production by GCs.

## Materials and methods

Except otherwise stated, all chemicals were obtained from Sigma (Sigma Chemical Co., St Louis, USA).

### Cell culture and stimulation

The human granulosa cell line KGN was established by Nishi et al., 2001 from a stage III granulosa cell carcinoma [12]. Human KGN cells were cultured as previously described in DMEM (Dulbecco's Modified Eagle Medium), 10% FCS (Fetal calf serum) and a final concentration of 100 units/ml of penicillin and 100 µg/ml of streptomycin. (1% Pen/Strep) at 37 °C and 5% CO_2_ [13, 14]. Cell passages were 3–5.

The OvCar-3 cell line was cultivated in DMEM, 10% FCS (Fetal calf serum) and 1% Pen/Strep at 37 °C and 5% CO_2_. Cell passages were 2–5.

In brief, cells were seeded in 6-well plates at a density of 1 × 10^5^ cells per ml. After 24 h, the medium was discarded and the remaining cells were washed twice with PBS and new media were added. Stimulation was carried by adding Forskolin to a final concentration of 1, 10, 50, 100, 200 µM, respectively. After 72 h, the supernatants were collected and stored for hormone quantification. In the case of co-culturing 5 × 10^4^ of each cell line were seeded.

For cell passaging media was discarded and cells were treated with 3 ml Trypsin/EDTA solution containing 0.5 g trypsin and 0.2 g EDTA for 3 min at 37 °C, 5% CO_2_ after washing with PBS. The reaction was stopped by adding 2 ml of media containing 10% FCS. The detached cells were centrifuged for 5 min at 800 g. The media was discarded and new media was added to the cell pellet. The cell pellet was solved by gentle pipetting and cells were counted after Trypan Blue staining in a counting chamber.

### Hormone quantification

Cell culture media was collected after 72 h of treatment and kept frozen at − 80 °C prior to testing.

Enzyme-linked immunosorbent assays (ELISAs) for estrogen (17beta-estradiol) and progesterone were performed using an ELISA kit according to the manufacturer's instructions (IBL international, Hamburg, Germany). Essentially, 25 µl of each sample was added in duplicate to a 96-well ELISA plate and incubated for 1 h at room temperature. Unbound material was washed out three times with washing solution. After addition of substrate solution, plates were incubated for an additional 15 min and stop solution was added. Absorbance (OD) was determined using a microplate reader (LumiStar Optima, BMG, Kumberg, Austria) at 450 nm.

Intra-assay variability and inter-assay variability for the progesterone ELISA ranged from 5.4 to 6.99% CV and from 4.34 to 9.96% CV, respectively. Intra-assay variability and inter-assay variability for the estrogen ELISA ranged from 8.70 to 90.23% CV and from 6.87 to 14.91% CV, respectively.

### Statistics

The results of the hormone quantification experiments have been analyzed using ANOVA for independent samples followed by Tukey's HSD test. Differences were considered statistically significant at *p* < 0.05. Statistical analyses were performed at the statskingdom website at https://www.statskingdom.com.

## Results

**Fig. 1:**
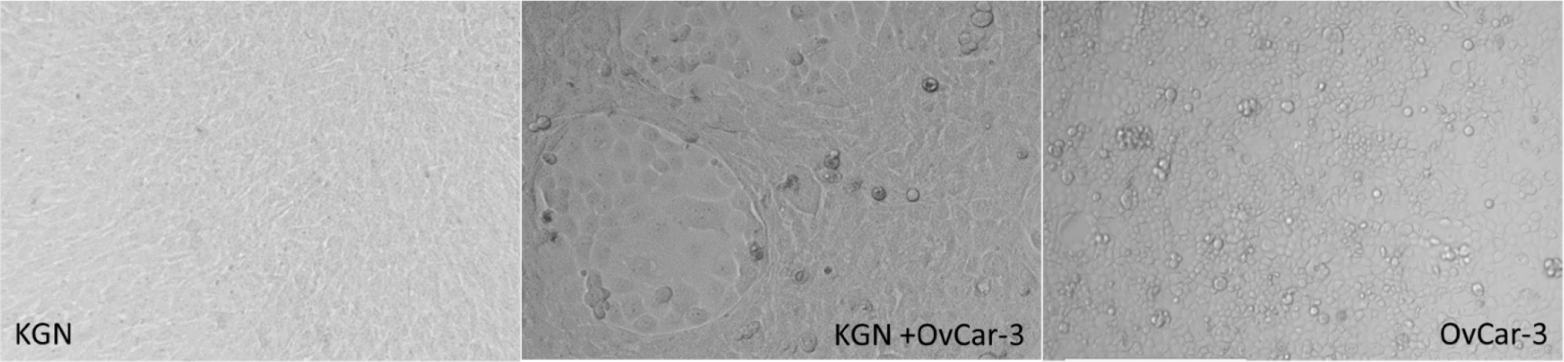
Morphology of KGN and OvCar-3. Representative pictures of fibroblastic KGN cells (left), KGN cells and OvCar-3 cells in co-culture (middle) and cubic OvCar-3 cells (right). OvCar-3 cells form island-like structures surrounded by KGN cells in the co-culture experiment. All cells were unstimulated

**Fig. 2:**
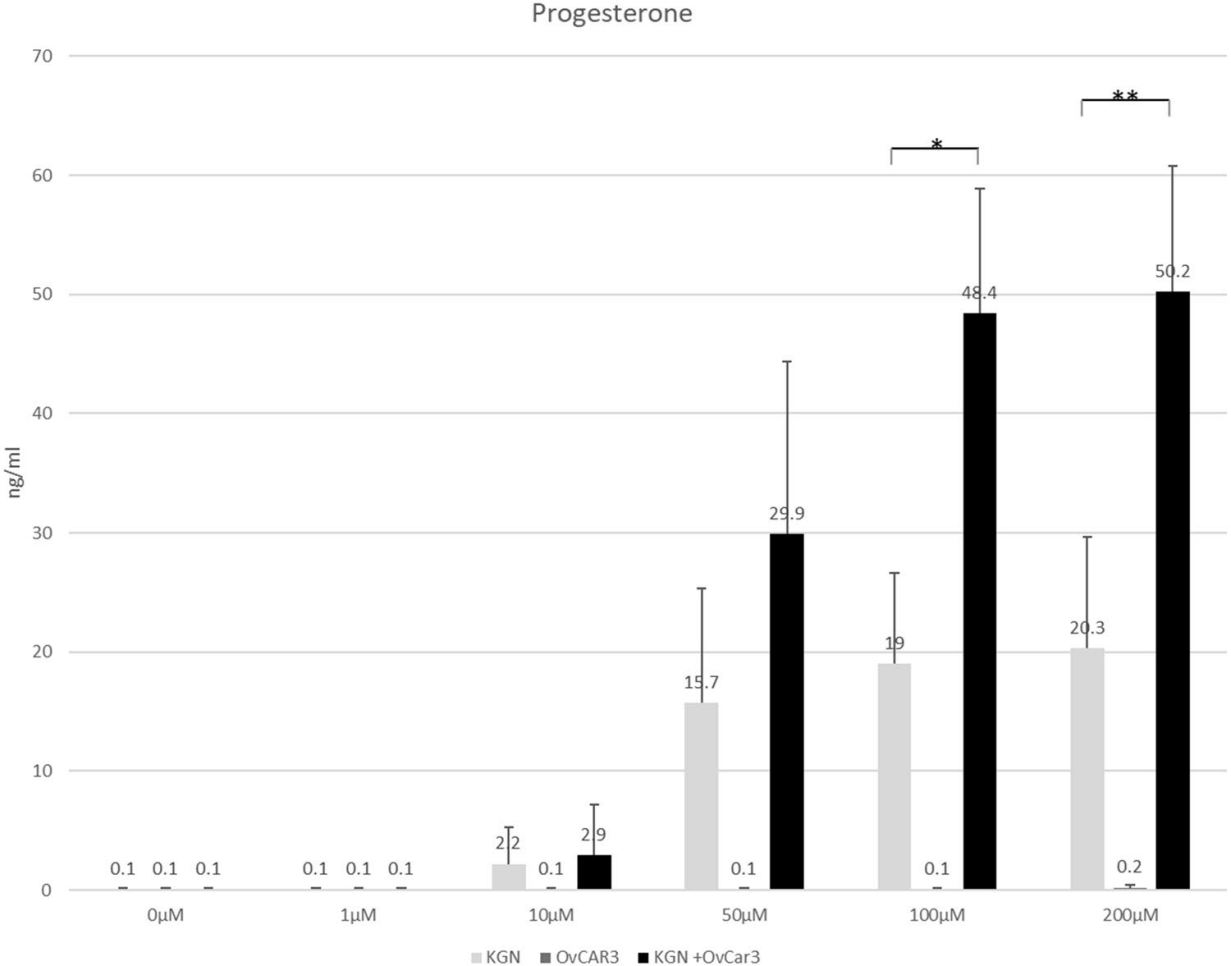
Progesterone production. Effect of various concentrations of forskolin (1 µM to 200 µM) on the progesterone production of KGN, KGN + OvCar-3 and OvCAR-3 cells. All cultures were carried out for 72 h. Each bar represents mean ± STD. Individual experiments were repeated at least 4 times (*n* = 4). *Significant at a *p*-level < 0.05, **significant different at a *p*-level < 0.01

To investigate the effect on steroid hormone production by co-culturing a granulosa cell line and an epithelial carcinoma cell line, both cell lines were cultured under standard cell culture conditions separately and in a 1:1 ratio for 72 h. Morphologically, Fig. 1 shows that OvCar-3 and KGN together build distinguishable areas and the cell lines do not completely intermix. To investigate the hormone production capacity of the cells after culturing, we stimulated the cells with increasing amounts of forskolin, a cyclic adenosine monophosphate (cAMP)-inducing agent. Although OvCar-3 cells were not known to produce progesterone, we assessed this possibility by measuring progesterone concentration after stimulation with increasing amounts of forskolin. As demonstrated in Fig. 2, the stimulation of the OvCar-3 cells has no detectable effect including a concentration of 200 µM forskolin. Stimulation of the human granulosa cell line KGN demonstrates a concentration-dependent increase in progesterone production from 2.2 ng/ml at a progesterone concentration of 10 µM to an amount of 20.3 ng/ml at a concentration of 200 µM forskolin. When we cultivated both cell lines in a co-culture and a ratio of 1:1, we observed an increase in progesterone production from 2.8 ng/ml at a concentration of 10 µM forskolin up to 50.2 ng/ml at a concentration of 200 µM forskolin. We would like to point out that the amount of progesterone-producing KGN cells in these co-culture experiments is about half the amount of the KGN single-culture experiments. Additionally, we performed the same series of experiments and determined the estrogen production of both cell lines. However, significant amounts of estrogen was not detectable (data not shown).

## Discussion

In our study, we were able to show that the KGN–OvCar-3 co-culture system is a suitable model for demonstrating a stimulatory paracrine effect of co-culturing these two ovarian cell lines on progesterone production.

The progesterone synthesis of the human granulosa cell line KGN is mediated by a cAMP-triggered mechanism involving protein kinase A (PKA) dependent pathways [15]. Therefore, it is suitable to increase progesterone production either by increasing the amount of cAMP in the cells or by stimulating the enzyme adenylate cyclase with forskolin as we did in our study.

The progesterone amounts measured in our experiments for KGN cell culture after 72 h and stimulation with 200 µM forskolin are in the same physiological range as the values reported by Nishi et al., after (Bu)2cAMP stimulation (20.3 ng/ml vs. 15 ng/ml) (Nishi, Yanase et al. 2001). In the co-culture experiments together with OvCar-3 cells, however, we reached values up to 50.2 ng/ml, which corresponds to an increase by a factor of about five, taking into account that only half of the KGN cells have been seeded. A modulatory effect on cell viability and function by co-culturing different cell types was shown by Lancaster et al., for stem cell based organoid models [16]. In addition, a form of self-organization of granulosa cells and different other cells has already been described by Qiu et al. [17] as well as our group [18, 19]. De Souza and colleagues have shown that mesenchymal stem cells (MSC) increase viability and hormone production capacity of islets cells [20] and Jiang and colleagues reported a regulatory effect by endothelial cells on microglial cells [21]. A stimulatory effect by co-culturing on cell migration of cancer cells and on ovarian differentiation was reported by Dogan et al. [22] and Mackay et al. [23]. Our data are in line with results of other authors that co-cultivation of different cell types from ovarian origin can have a significant effect on hormone production. For example, the data of Qiu et al. have shown in in vitro experiments that co-culturing ovarian cortical stromal cells with primary granulosa cells derived from goat ovaries can stimulate both progesterone synthesis and cell proliferation.

Human granulosa cells are the site of hormone production in the ovary and play a crucial role in oocyte development trough interaction with a variety of other cells and cell types, resulting in a coordinated synergistic physiological process. It is justifiable to state that the failure of a small part of this system can lead to the breakdown of the entire physiological network. Our simple in vitro model of cooperation of granulosa cells and epithelial ovarian cells in hormone production supports the hypothesis that also in ovarian tissue, the cell types adjacent to the granulosa cells make an important contribution to hormone production and, thus, also to functionality. However, a restriction of the validity of our model is that we are dealing with two cell lines and therefore a generalization to the in vivo situation seems to be possible only to a limited extent. Our model uses cancer cells, not primary cells and cannot fully describe the complex interactions, which occur in vivo.

In the special situation of cryopreservation and re-transplantaion of ovarian tissue, fundamentally different cell types must cope with the stressors of freezing and the potentially toxic properties of the cryoprotective solutions. Therefore, special care should be taken in account to ensure that all cell types of the tissue are viable again after warming to return to full functionality after re-transplantation. Cell types that were most severely damaged by the procedure used may be decisive for the success or failure of the therapy in terms of different vitality.

## Conclusion

Our model seems to be a simple and “easy to handle” model to study the effects of different cell types on the ability of human granulosa cells to produce progesterone. Furthermore, it highlights the relevance of survival of all cells in endocrine tissues after cryopreservation and subsequent re-transplantation.

## Data Availability

Data available on request from the authors.
